# In the shadow of Graves’ disease: a qualitative interview study of patients’ experiences conducted at a secondary referral centre in Sweden

**DOI:** 10.1136/bmjopen-2024-098238

**Published:** 2025-11-16

**Authors:** Agneta Lindo, Sara Alsén, Andreas Fors, Helena Filipsson Nyström

**Affiliations:** 1 Department of Endocrinology, Sahlgrenska University Hospital, Gothenburg, Sweden; 2 Institute of Medicine, Sahlgrenska Academy, University of Gothenburg, Gothenburg, Sweden; 3 Wallenberg Center of Molecular and Translational Medicine, Region Västra Götaland, Gothenburg, Sweden; 4 Centre for Person-Centred Care (GPCC), Sahlgrenska Academy, University of Gothenburg, Gothenburg, Sweden; 5 Institute of Health and Care Sciences, Sahlgrenska Academy, University of Gothenburg, Gothenburg, Sweden; 6 Research, Education, Development and Innovation, Region Västra Götaland, Primary Health Care, Gothenburg, Sweden

**Keywords:** Person-Centered Care, Patient-Centered Care, Fatigue, Thyroid disease

## Abstract

**Abstract:**

**Objective:**

Graves’ disease (GD) is the most common form of hyperthyroidism in Sweden with an incidence of 21/100 000 individuals, the majority of whom are women of working age. GD can be overwhelming for the affected patient. A way to improve health outcomes is to better understand patients’ experiences of their illness. We therefore aimed to explore patients’ experiences of GD during the initial phase of the disease.

**Design:**

A qualitative study based on semistructured interviews was conducted and analysed using qualitative content analysis, following Graneheim and Lundman’s description of the method.

**Setting:**

The study was carried out within specialised care at the secondary level in a Swedish healthcare context.

**Participants:**

15 patients (12 women and 3 men; aged 29–74 years) within the first 3 months after GD diagnosis were included in the study.

**Results:**

Being affected by GD means facing a range of new and often incomprehensible symptoms contributing to an experience of change in one’s personality. In contact with healthcare, they experienced challenges such as an overwhelming amount of information, a lack of energy, and feelings of being a burden. These factors were described as having a negative impact on daily life, well-being, and psychological and psychosocial functioning. The participants highlighted the need to be listened to, to receive tailored information, to have continuous contact, and to have fatigue and other symptoms more thoroughly addressed.

**Conclusion:**

The findings indicate that symptoms have a significant impact on patients with GD, influencing their care experience, information processing, decision-making abilities, and daily functioning. The application of person-centred care can be one way to support patients with GD, as it facilitates a collaborative approach and enhances the comprehension of each patient’s needs and resources. By acknowledging the patient’s experiences, situation, and expectations, as well as the comprehensive impact of the disease, and by modifying support strategies, patient well-being and health outcomes may be significantly improved.

**STRENGTHS AND LIMITATIONS OF THIS STUDY**

- The study explores an area with limited research: patients’ experiences of Graves’ disease in its early stages.
- A diverse sample of women and men, varying in gender, age, ethnic background, education and time from diagnosis, was recruited to ensure broad representation.
- The study reveals a pattern of patients’ views on their illness and support needs, providing valuable insights for improving care.

- The study may have unintentionally favoured participants who had the strength to engage in interviews, potentially excluding those who were too unwell to contribute.
- The study included narratives from 12 participants; although small sample sizes are common in qualitative research, the limited number may reduce the extent to which the findings apply to other contexts.

## Introduction

 Graves’ disease (GD) is the most common form of hyperthyroidism with an incidence of 21/100 000 individuals.^1 2^ GD mostly affects working-age women, causing symptoms like sweating, tremors, fatigue, palpitations, tachycardia, and cognitive impairment.^3^ The latter can make information processing challenging during the hyperthyroid phase. Impatience, fear, and stress intolerance are also integral parts of GD symptomatology, especially in the acute phase.^4^ Despite antithyroid drug (ATD) treatment normalising thyroid hormone levels, approximately 25% of patients report persistent symptoms such as fatigue,^5^ which may affect overall well-being^6^ and reduce health-related quality of life (HRQoL) with lasting impacts even after a year.^7^ Furthermore, many patients need sick leave, emphasising the importance of early clinical assessments to improve outcomes.^8^

Research indicates that, compared with controls, patients with GD have higher levels of mental fatigue, depression, anxiety,^3 9^ and an increased risk of hospitalisation and medication for mental health issues,^10^ emphasising a need for better understanding and support. In an interview study (with an average GD treatment duration of 6 years), patients reported feeling disconnected from themselves and perceiving their body and mind as separate entities.^11^ Studies involving patients with other conditions experiencing symptoms similar to those of GD, such as stress-related exhaustion, emphasise the importance of patients being listened to, taken seriously,^12 13^ and respected by their support networks.^12^

In Sweden, patient involvement is underscored by Swedish law,^14^ emphasising personal preferences and circumstances. However, a knowledge gap persists regarding how patients with GD perceive their condition and the care they receive,^15^ a concern also highlighted in Swedish national guidelines.^16^ Therefore, to gain a better understanding and discover potential ways to improve care, the aim of our study was to explore patients’ experiences of GD during the initial phase of the disease.

## Methods

### Study design

This is an exploratory interview study of patients within 3 months of their diagnosis with GD. Data were analysed using qualitative content analysis, following Graneheim and Lundman’s description of the method.^17^,^19^

### Participants and setting

Participants were recruited from the thyroid unit at the Department of Endocrinology (Sahlgrenska University Hospital, Gothenburg, Sweden). Care and treatment of GD are performed by endocrinologists at a secondary referral level in Sweden. General practitioners often diagnose hyperthyroidism and prescribe beta-blockers, while referring patients for ATD treatment and discussion of long-term treatment strategies. Private endocrinologists are few, and most care is provided by tax-financed healthcare. The first author, a registered nurse, conducted a screening of all patients diagnosed with GD between July 2021 and February 2022. We ensured a strategic sample with variation in gender, age, ethnic background, education, and time from diagnosis. GD was defined by overt hyperthyroidism (high level of free thyroxine (fT4) with reference range: 9.8–17 pmol/L), low thyroid-stimulating hormone (TSH) and high levels of TSH-receptor antibodies (reference: <1.8 IU/L). Median fT4 was 27 pmol/L (range: 20–36 pmol/L) at diagnosis. In addition, the following inclusion criteria were applied: age >18 years, patients within the first 3 months after GD diagnosis, ability to understand and communicate in Swedish, and having a mental capacity to participate in an interview study. The reason for including patients within the first 3 months after diagnosis was that this period represents a sensitive phase, when hormone levels are often elevated and treatment is being adjusted. Understanding their experiences at this stage may help identify how best to support them in navigating healthcare and feeling secure. A total of 27 patients were initially contacted by telephone and informed about the study. 12 patients declined, and 15 agreed to participate (12 women and 3 men, median age 46 (range: 29–74) years) (table 1). Three of the 15 participants were on sick leave at the time of the interview, and two had an ethnic background other than Swedish.

**Table 1:** Characteristics of the participants

	No. (%)
Sex	
Female	12 (80.0)
Male	3 (20.0)
Age (years)	
18–30	2 (13.3)
31–40	3 (20.0)
41–50	6 (40.0)
51–65	0
>65	4 (26.7)
Civil status	
Living alone	4 (26.7)
Living with a partner	11 (73.3)
Time from diagnosis to interview (weeks)	
3–6	9 (60.0)
7–9	3 (20.0)
10–12	3 (20.0)
Highest level of education	
Compulsory school	0
Secondary school	3 (20.0)
Vocational college	1 (6.7)
University	11 (73.3)

### Data collection

Semistructured, face-to-face interviews were conducted in a private room at the Thyroid Unit between October 2021 and April 2022 by the first author using an interview guide (for the English translation see online supplemental file l). The aim of the study was explained to the participants, and the opening question was ‘What is your experience of being affected by GD?’ Other questions included ‘How do you manage the disease today?’, and ‘What kind of support did you expect from the healthcare system when you became ill?’ The interview guide was developed by the research team, with the involvement of a patient representative and some patients who piloted the guide and provided constructive feedback that informed subsequent revisions and refinements. The interviews lasted 26–50 (median: 39) minutes and were audio-recorded and later transcribed verbatim by the interviewer or a secretary. Follow-up questions such as ‘Could you please tell me more about that?’ or ‘Can you give me an example?’ were asked throughout the interview to obtain comprehensive responses. The interviews were conducted until the authors considered the research questions to be fully addressed.

### Patient and public involvement

A representative of the National Thyroid Patient Organisation was involved in the study design, development of the interview guide, and provided input on the patient information, study protocol, and the methodology employed in the interviews.

### Data analysis

Content analysis uses empirical data to describe and interpret a phenomenon, enhancing understanding. This method comprises several stages. Initially, the interviews were reviewed on multiple occasions to gain an overall understanding of their content. The text was divided into meaning units, encompassing words, sentences, and phrases related to the study’s aim. These units were condensed and assigned codes, which were interpreted, compared for similarities and differences, and sorted into four categories with subcategories. To ensure trustworthiness and a sincere interpretation of the data, all authors, comprising registered nurses, an endocrinologist, and an occupational therapist student, discussed their pre-understanding from previous care and research (e.g., in hyperthyroidism and GD). All authors conducted repeated reviews of the text and interviews, considering alternative interpretations until consensus was reached. The quotations in the article were translated into English by the authors, and we reviewed the translations to ensure that the original meaning and nuance were preserved, rather than simply providing a word-for-word rendering.

## Results

**Figure 1:**
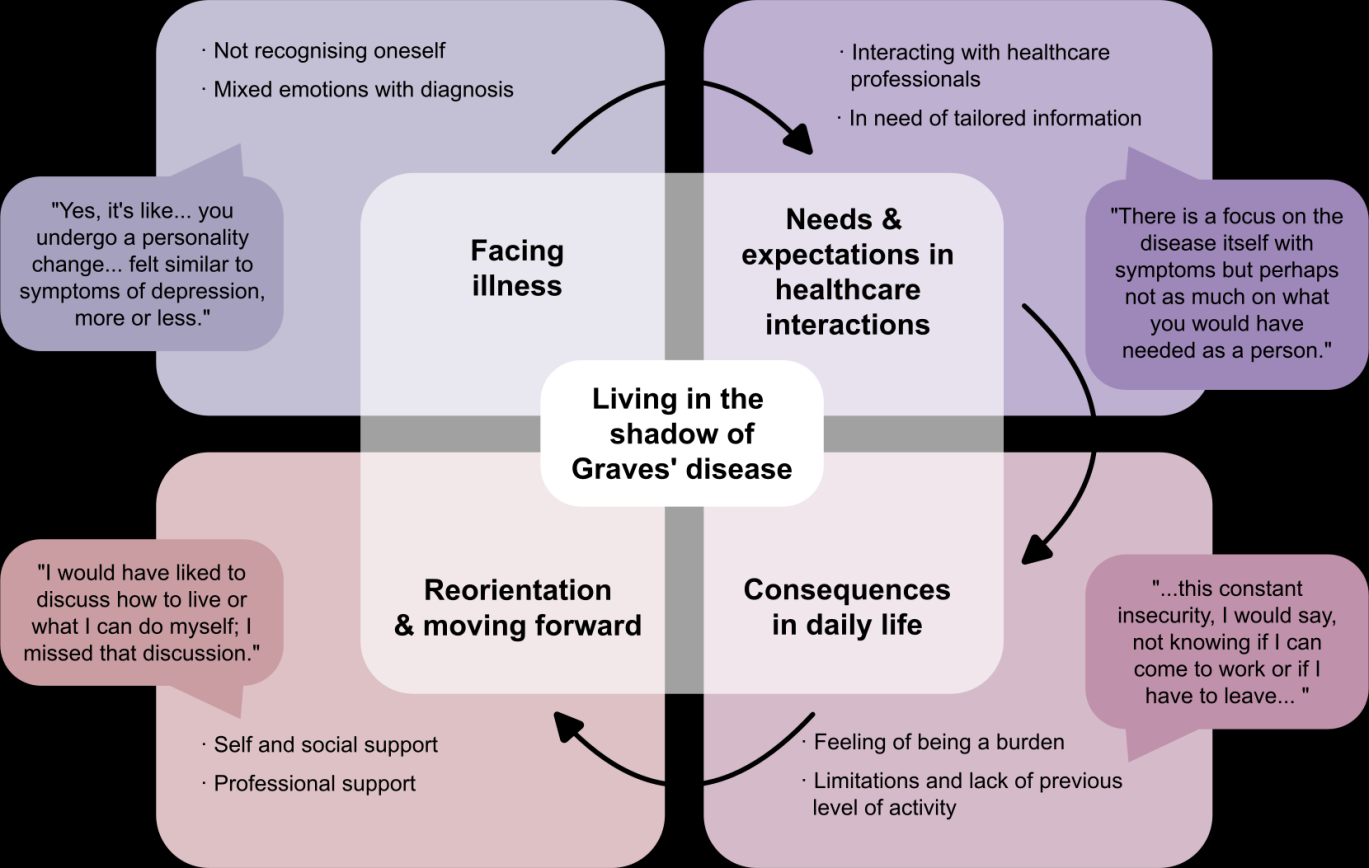
Overview of the theme with related categories and subcategories identified in the qualitative analysis.

The theme, ‘Living in the Shadow of Graves’ Disease’, was revealed through the analysis of the interviews and reflected in four categories: (1) facing illness, (2) needs and expectations in healthcare interactions, (3) consequences in daily life, and (4) reorientation and moving forward. Each category and its respective subcategories are presented in figure 1 and illustrated by quotes assigned to participants by anonymous numbers.

### Facing illness

This category describes how the diagnosis and symptoms related to GD were experienced by the patients. Facing illness was expressed in two subcategories: ‘not recognising oneself’ and ‘mixed emotions with diagnosis'.

#### Not recognising oneself

Facing GD presented significant challenges for patients, who experienced a range of new and often incomprehensible symptoms. These included memory lapses, fatigue, irritability, anxiety, fear, depression, heightened sensitivity, stress, lethargy, sweating, tremors, and a reduced capacity for patience, often occurring simultaneously and persisting over an extended period. The initial symptoms of GD were described as diffuse, making it difficult to identify the underlying cause. One participant recalled, “I was trembling so much. I said, ‘Yes, but I don’t feel… I don’t feel so stressed.’ It’s strange that I can’t… I have no control over my body.” (Participant no. 11).

The resemblance of symptoms between GD and other diseases resulted in confusion and heightened the probability of misinterpretation. The participants found it challenging to determine whether their symptoms were a result of life stressors, such as family or work pressures, or indicative of an underlying medical condition. All these symptoms contributed to the patients’ feeling as if they did not recognise themselves. “Yes, it’s like… you undergo a personality change… felt similar to symptoms of depression, more or less.” (Participant no.6).

#### Mixed emotions with diagnosis

There was a period of waiting to receive help and answers regarding the cause of their condition. In some cases, the time between the onset of the symptoms, diagnosis, and subsequent treatment was perceived as long. “There was a time when I was just waiting; I got worse day by day…” (Participant no. 1). Some patients described a worsening of their symptoms and found it difficult to find an appropriate source of help. This situation was particularly distressing and had a significant impact on the participants’ well-being, feelings of safety, and uncertainty regarding the origin of their symptoms.

Receiving a diagnosis triggered mixed emotions among the participants. For some, the news brought a sense of relief, explaining their troubling symptoms. “I felt an incredible relief that I wasn’t going crazy in my head.” (Participant no. 7). Others, however, struggled with shock and disbelief because the diagnosis came as a surprise. “It came very suddenly, just like a lightning bolt from a clear sky.” (Participant no. 14).

Additionally, there was a pervasive fear of developing other autoimmune diseases coupled with lingering uncertainty about the long-term persistence of their symptoms. This mixture of relief, fear, and uncertainty made the diagnosis a profound and life-altering experience.

### Needs and expectations in healthcare interactions

This category encompasses the experience of encountering and receiving information from healthcare professionals (HCPs) and was formulated in two subcategories: ‘interacting with HCPs’ and ‘in need of tailored information’.

#### Interacting with HCPs

Patient experiences of interacting with HCPs varied considerably. Some patients felt warmly welcomed, listened to, and acknowledged by HCPs, while others felt dismissed and overwhelmed by the medical approach. Overall, there was a strong focus on medical aspects, such as test results, vital signs, and medication. Despite the welcoming atmosphere, patients noted a lack of attention to psychosocial factors, such as how their well-being affected their everyday life. Some participants described that they were not asked about how they felt, but rather whether they exhibited specific symptoms or signs. They perceived that there was a lack of opportunity to discuss the impact of the disease on their life circumstances. “I would have liked to have been asked how I felt; perhaps, how I felt in this as well.” (Participant no. 7).

There was a lack of consistency in the frequency of visits to the same physician, with patients often seeing different HCPs at each appointment. This made it challenging for many patients to establish a relationship with physicians, making it difficult to identify a point of contact for follow-up questions. “The contact with at least four different doctors, it can be… may not be optimal…” (Participant no. 1).

Additionally, there was confusion about how to contact HCPs for enquiries. This was further complicated by instances where initial contacts were handled through a service centre, which forwarded matters for assessment. This led to concerns about response times and the accuracy of information being transferred. Several participants expressed a desire for consistent support and dialogue.

#### In need of tailored information

The participants experienced a range of responses to the information provided regarding their condition, treatment options and sick leave. Some described feeling overwhelmed by the quantity of information provided and expressed a preference for a greater focus on their personal well-being and the practical implications of their condition. “There is a focus on the disease itself with symptoms, but perhaps not as much on what you would have needed as a person.” (Participant no. 6).

For some, the sheer volume of information presented made it challenging to fully comprehend the various treatment options. “It was a bit too much about the different options… I just want to get well…” (Participant no. 3).

Regarding sick leave, some participants experienced a lack of clarity regarding its significance, which initially led to reluctance despite the necessity of it for recovery. “I was offered sick leave but did not take it… I do not think I realised how I felt.” (Participant no. 6).

The presence of a family member or friend during medical visits was found to facilitate information retention and provide emotional support. The participants experienced difficulties in making decisions regarding their treatment options, communicating with the clinic, and understanding their role as patients. It was expressed that there is a need for more transparent expectations and communication regarding care plans, as the initial visits left the patients feeling uncertain about the subsequent steps. “I don’t know if I’m going to see anyone again. I guess I will.” (Participant no. 8).

The participants expressed a need for consistent support, tailored information, and clearer communication to effectively navigate their care journey. “You don’t find out as a patient what is expected of me.” (Participant no. 2).

### Consequences in daily life

This category encompasses the experience of how the illness impacted patients’ daily life and is described in two subcategories: ‘feelings of being a burden’ and ‘limitations and lack of previous activity level’.

#### Feelings of being a burden

Participants expressed that they were placing burdens on their families due to the impact of the disease. Partners had to assume additional responsibilities, especially during times when the participant’s abilities were significantly restricted. “My partner had to take on a huge load ever since then. Now, I manage to take the dog out for a quiet walk, but there was a period when I couldn’t do anything, and she had to carry the whole load.” (Participant no. 1).

Several participants who had children described feelings of guilt over their reduced ability to spend quality time with them compared with before. They mourned the loss of their previous level of engagement and interaction, which had been a significant and fulfilling part of their lives. This guilt was compounded by the awareness that their illness had inadvertently impacted their children’s daily routines and emotional well-being. “I’m a little sad because they (the children) stress me out.” (Participant no. 4).

GD also affected work life, with some participants choosing to disregard medical advice and continuing to work, while others were either partially or completely absent from work due to illness. The uncertainty about their ability to work posed a significant challenge. “This constant uncertainty, not knowing if I can go to work or if I have to leave…” (Participant no. 6). One participant expressed frustration over a lack of understanding from their workplace regarding the effects of the disease. “I have to explain things to my workplace; I feel it’s hard; I’m afraid to come back; there are expectations…” (Participant no. 5).

#### Limitations and lack of previous level of activity

Many participants described that the disease had imposed significant limitations on their activities, leading to the loss of past hobbies, family activities, and planned events due to their reduced abilities. “I can’t do anything by myself, can’t participate in activities or do the things I like, such as cycling, which was a big part of my leisure time. I can’t even keep up with the kids.” (Participant no. 3). Some participants encountered challenges in initiating activities because of persistent fatigue or a perceived inability of their body to manage the demands. “I sat like in a bubble. Cotton round my whole head as if I wasn’t there.” (Participant no. 12).

Nevertheless, some participants found it challenging to refrain from exercise at the outset of their illness, particularly when hormones were elevated. Exercise had previously served as an effective coping mechanism for managing the stressors of daily life. For many, who were already engaged in regular physical activity, it was challenging to distinguish between the need for illness management, daily life activities, and recovery. The difficulty in balancing these aspects often led to confusion about how best to adjust their routines to handle their changing health needs. “Absolutely the biggest challenge for me is that I have not been able to start training; it is something that is really a challenge; it takes almost more on the psyche than the disease does I think.” (Participant no. 9).

### Reorientation and moving forward

After coming to terms with the diagnosis, the participants began the process of reorientation and moving forward. This category was formulated into two subcategories: ‘self and social support’ and ‘professional support’.

#### Self and social support

The participants described their own strategies for managing the disease, indicating that their lives could no longer continue as before. They sought out new activities that offered a sense of calm and concentration amidst the chaos. Over time, several participants came to accept the new situation of living with the disease, emphasising that acceptance was important for making progress. Some personal strategies included the delegation of tasks to partners, the acknowledgement of personal limitations and the prioritisation of self-care. As one participant observed, “I have initiated a knitting practice to facilitate relaxation and sustained attention on a single task, and it has proven to be an efficacious approach.” (Participant no. 11).

In addition to personal strategies, family was identified as a vital source of support. Many participants relied on their relatives for both practical assistance and emotional encouragement during challenging periods. For example, one participant stated, “My mother is a source of great support; if I require childcare at this time, she is willing to provide it.” (Participant no. 7).

For many participants, connecting with others who face similar challenges was an invaluable source of support, providing reassurance and a sense of friendship.

#### Professional support

Although participants appreciated the support they received during initial meetings, they expressed a need for continuous professional support. “I experienced a lot of support during the initial meeting, but since then, I haven’t had any; I haven’t reached out either.” (Participant no. 3).

There was a desire for comprehensive support beyond medical consultations, including discussions on managing daily life and accessing therapy. *“*I would have liked to discuss how to live or what I can do myself; I missed that discussion.” (Participant no. 4).

Several participants expressed dissatisfaction with the lack of support and tools available to manage their condition. They emphasised the importance of therapy, stress management and nutritional guidance. “Some type of therapist to juggle with and deal with the stress would be suitable.” (Participant no. 9).

They also wanted help in bridging the gap between medical information and its impact on work and family life, underlining the importance of clear communication and tailored support. “Sitting down and talking to someone about it, even if it’s just once, could be beneficial.” (Participant no. 8).

Thus, the participants sought comprehensive support encompassing emotional, practical, and informational aspects to effectively manage life with their illness.

## Discussion

Being affected by GD was found to be a profound challenge by the participants, significantly limiting their ability to function as they had before the onset of the disease. The interviews provided comprehensive insights into their first 3 months following diagnosis, revealing that GD affects patients far beyond its immediate physical symptoms. Our interpretation of living with GD can be understood as living in the shadow of the disease due to its psychological impact. The disease was described as affecting their personality, with some feeling that they no longer recognised themselves, which in turn influenced their daily lives.

Furthermore, the assumption that all individuals have access to adequate social support may not always hold true, highlighting the importance of addressing these aspects in care and follow-up.

In contact with healthcare, patients described difficulties absorbing all the information and recommendations conveyed by the HCPs. Our results indicate that patients who experienced psychological issues from the disease had feelings of inadequacy and an inability to meet expectations. Given these findings, it is important to consider the nature of healthcare appointments, which were primarily medically oriented. The participants expressed a greater need for support in managing their psychological well-being rather than just discussing their medication. Follow-ups can therefore provide reassurance, support patients in managing their treatment and strengthen continuity of care. This suggests that a more person-centred approach to care might be beneficial for patients with GD. Person-centred care (PCC) emphasises the importance of knowing the patient as a person by listening to the patient’s narrative and co-creating their care. This approach considers both the perspective of the patient and the expertise of the HCPs to make informed decisions. It also involves a collaborative approach to planning and treatment to achieve the best possible outcome.^20^ Studies evaluating the effects of PCC have demonstrated favourable outcomes, such as improved self-efficacy, reduced symptom burden, shortened length of hospital stay, cost savings, and increased satisfaction with the care.^21^

Therefore, HCPs need to listen more thoroughly and provide information in a step-by-step manner, as our findings show that patients have difficulty retaining excessive information. This could help reduce patient stress levels, enhancing their ability to absorb information and, furthermore, enabling them to express how they are feeling. In turn, this PCC approach might facilitate a shared understanding between the patient and the HCPs, allowing interventions to be more tailored to the specific needs and resources of each patient.^20 21^ According to Swedish law,^14^ HCPs have a duty to recognise and address patient needs and collaboratively plan their care based on their support requirements.^14^ When patients experience being in the shadow of GD, PCC would address many of the difficulties expressed by participants when facing illness and their needs and expectations during contact with healthcare.

In our study, patients with GD described significant fatigue, which negatively impacted their daily lives and work performance, making it harder to engage in social activities and maintain their previous level of occupational functioning. This study, the first to explore patients’ experiences of GD through interviews in the initial phase of their disease, confirms that fatigue is a core symptom, which is consistent with previous quantitative research.^3^ Fatigue was highlighted by participants as a particularly challenging symptom, underscoring its importance for HCPs to recognise as it contributes to broader difficulties for patients.

In a recent study, 89% of female patients with GD and severe hyperthyroidism exhibited mental fatigue, which persisted in 38% of them after 15 months.^3^ Mental fatigue is a syndrome that includes irritability, difficulties with concentration and memory, sleep disturbances, tearfulness, and fatigability, which may be seen in many conditions.^3^ Mental fatigue is a troublesome symptom,^22^ and it is sometimes combined with anxiety and depression.^3^ Hyperthyroidism is viewed as a temporary state, but many studies indicate long-term cognitive impairment and reduced HRQoL.^45 7 23^,^30^ Although patients with GD have normal results on cognitive tests,^3^ they experience cognitive impairment in daily life^3^ with difficulties in taking part in everyday activities^26^,^30^ and lower work ability.^25 31 32^

This is consistent with our results, where the patients experienced fatigue, lack of self-recognition, feelings of being a burden, limitations in activities and a sense of loss. While physical symptoms associated with GD are well-documented, research on mental health outcomes is limited and mental symptoms are often overlooked by HCPs. The national Swedish guidelines for hyperthyroidism (2023) recommend rehabilitation of mental fatigue if it persists despite regaining euthyroidism,^16^ as there are common strategies to be applied regardless of cause.

Patients often experience persistent feelings of unrecognised struggle, even long after diagnosis.^11^ They also describe a sense of disconnection between their body and mind along with profound exhaustion.^11^ A comparison of the aforementioned study with our own, conducted at an earlier stage in the course of the disease, indicates that the symptoms persisted even after a long period of treatment. This underscores the importance of early identification of the patient group to provide adequate support to assist patients in understanding their condition and in accepting their circumstances. Managing symptoms and communicating them to others^11^ can be challenging, particularly when they are not medically confirmed. It has proven crucial for their self-understanding that HCPs can recognise, understand and acknowledge a patient’s symptoms.^33^

Research has demonstrated that the use of patient-reported outcome measures (PROMs) can enhance symptom management and improve patients’ HRQoL.^34^ PROMs may provide a structured way for patients to communicate their experiences, symptoms, and concerns, while patient-reported experience measures (PREMs) capture their experiences of care.^35^ For patients with GD, the Thyroid-related Patient-Reported Outcome questionnaire may be used,^36^ which has been shown to be responsive to clinical changes.^34^ This questionnaire and the Mental Fatigue Scale recognising the mental fatigue^37^ are validated and clinically relevant instruments to recognise patient symptomatology, as recommended by a Danish study^34^ and national Swedish guidelines.^16^

### Methodological limitations

Although the sample size was small (n=15), this is typical for qualitative research, which seeks depth and variation in participants’ experiences rather than generalisable findings. Interviews continued until the data were deemed sufficient to address the research question; however, the limited sample may influence the transferability of the findings to broader contexts. Similarly, context-specific factors could have influenced participants’ accounts. Despite these limitations, the consistent patterns across diverse participants suggest that the study provides meaningful insights into experiences of the illness, while acknowledging that cultural and contextual differences should be considered when applying the findings to other settings.

### Conclusion and clinical implications

Our findings highlight the need to strengthen PCC in clinical practice to better meet the needs and expectations of patients with GD. GD can be experienced as a change in personality that affects daily life more than expected, often accompanied by low energy, overwhelming information, and feelings of being a burden. The patient’s narrative is central to PCC, which emphasises understanding each patient’s unique experiences and establishing a partnership between the patient and HCPs, which has been shown to improve health outcomes. PROMs and PREMs can complement the patients’ narrative by providing structured insights into symptoms, quality of life, and experiences of care, thereby facilitating meaningful discussions, tailored care and engaging patients in decision-making and care planning. Prioritising both physical and mental well-being remains essential for optimising care for patients with GD. Future follow-up interview studies at a later stage may add useful complementary data.

## Supplementary material

10.1136/bmjopen-2024-098238 online supplemental file 1

## Data Availability

No additional data are available.
