# Supplementary material for: In the shadow of Graves’ disease: a qualitative interview study of patients’ experiences conducted at a secondary referral centre in Sweden
Source: BMJ Open. 2025 Nov 16;15(11):e098238. doi: 10.1136/bmjopen-2024-098238 (PMC12625911; doi:10.1136/bmjopen-2024-098238)
Supplement: online supplemental file 1 [file bmjopen-15-11-s001.pdf]

Interview guide: patient experiences of falling ill with Graves' disease

Onset of the disease

- What is your experience of being affected by Graves' disease?
  - What led you to seek medical care? How did you notice that you were not feeling well?
  - What were your thoughts about yourself, your family, and your surroundings at this stage?
  - How did you perceive the reactions of those around you (relatives, friends, coworkers) to your illness?

- How do you manage the disease today?
  - What are the largest challenges of having Graves' disease?
  - Has the illness brought anything positive?
  - What consequences has the illness had for your daily life (and work)?

- What knowledge did you have about the disease before becoming ill?
  - How do you view your knowledge of the disease now?
  - How did you acquire your knowledge about the disease?
  - How would you like to receive information about your disease?
  - How did you experience the information you received from healthcare about Graves' disease?

Healthcare support for Graves' disease

- What kind of support did you expect from the healthcare system when you became ill?
  - How did you experience the support from healthcare after you became ill?
  - Can you describe an instance that you consider a good and a less good encounter with healthcare?

- How do you manage the disease today?
  - What support do you use today (personal resources, social network, healthcare)?
  - What advice would you give to healthcare professionals regarding the treatment of patients with Graves' disease?
  - What do you think could be improved in the care for Graves' disease?
  - What do you think can be supportive and helpful after falling ill with Graves' disease?
  - What would optimal care for Graves' disease look like for you?
  - Is there anything you would like to add that we have not covered?
