# Supplementary material for: In the shadow of Graves’ disease: a qualitative interview study of patients’ experiences conducted at a secondary referral centre in Sweden
Source: BMJ Open. 2025 Nov 16;15(11):e098238. doi: 10.1136/bmjopen-2024-098238 (PMC12625911; doi:10.1136/bmjopen-2024-098238)
Supplement: Supplementary file 2 [file bmjopen-2024-098238.reviewer_comments.pdf]

PEER REVIEW HISTORY

BMJ Open publishes all reviews undertaken for accepted manuscripts. Reviewers are asked to complete a checklist review form and are provided with free text boxes to elaborate on their assessment. These free text comments are reproduced below.

ARTICLE DETAILS

Title (Provisional)

In the Shadow of Graves' Disease: A Qualitative Interview Study of Patients' Experiences Conducted at a Secondary Referral Centre in Sweden

Authors

Lindo, Agneta; Alsén, Sara; Fors, Andreas; Nystrom Filipsson, Helena

---

VERSION 1 - REVIEW

Reviewer 1

Name Riguetto , Cinthia Minatel

Affiliation Endocrinology Division, Department of Internal Medicine, Faculty of Medical Sciences, University of Campinas, São Paulo, Brazil.

Date 07-Jan-2025

COI None

The paper is interesting and generally well-written, but I'm concerned about the number of participants, which is relatively small. I suggest discussing it as a limitation of your research.

Reviewer 2

Name Cheetham, Timothy

Affiliation Newcastle University

Date 10-Mar-2025

COI None

The authors have conducted interviews in a small number of patients with Graves disease soon after diagnosis. The objective was to understand in greater detail the patient experience of their illness.

This is generally well written.

Was there anything in the study findings that were felt to be surprising? Could the study conclusions have been predicted / written before the study started?

If you select a group of patients then inevitably some will have been managed by health professionals who are more empathetic than others. Are the authors in any way surprised that some health professionals are more person centred than others?

The fact that health professionals should be patient- centred is not revolutionary I would argue and a key component of best practice.

Some patients will have had more profound disease (thyroid hormone excess) than others - to what extent do the authors think that disordered biochemistry / the underlying disease might have compromised the ability of patients to engage with health professionals, even in the context of a patient centred approach?

The patient's first interview occurred during the 1st few months after diagnosis-as such some individuals will have been euthyroid, some still hyperthyroid and some potentially hypothyroid on treatment. How much of an issue might this be when reflecting on patient responses?

I would argue that it is unrealistic for someone with disease like Graves disease to comprehend all the nuances of the condition following the 1st or even 2nd consultations. How important is access to advice and guidance / expertise between appointments?

The authors talk about coming to terms with the diagnosis-do you think this is possible within the space of 3 months?

I wonder if the data would be more meaningful and revealing if the interviews were repeated 6 months or 12 months later - thoughts please - maybe the patient's perceptions of (for example) interaction with health professionals would have changed by then as relationships evolve?

**Reviewer**	**3**
**Name**	**Almquist, Martin**
**Affiliation**	**Skanes universitetssjukhus Lund, Dept. of Surgery**
**Date**	**12-Jul-2025**
**COI**	**None**

Thanks for letting me review this well-written manuscript describing a qualitative study on patients' experiences of being newly diagnosed with Graves' disease at a single tertiary hospital in Gothenburg, Sweden.

Graves' disease, i.e. hyperthyroidism due to autoimmune production of TSH-receptor stimulating antibodies, causes thyrotoxicosis, is most common among young women, and remains a significant cause for morbidity in this group. Understanding patient experiences when diagnosed with this disease is important and the authors shall be commended for undertaking this study.

As the authors note, there are many studies on QoL during different stages of thyrotoxicosis and its treatment, but a paucity of qualitative interview studies. Qualitative studies, where patients' perceptions, experiences and emotions are explored in depth, can reveal important insights, that more traditional studies focussing on biological and medical outcomes might miss. Such insights can be very useful to help health care improve and evolve. Qualitative studies also potentially offer a richer and deeper understanding of patient experiences than classical quality-of-life (QoL) or patient reported outcomes (PROMS) questionnaires.

Here, the authors interviewed 15 patients with newly diagnosed Graves' disease and interrogated them about their feelings about their disease, how they experienced the care that was given, how the disease affected their daily life and how they coped with living with the disease.

The paper reads very well and is written in concise and fluent English. I enjoyed reading it. Although I am no expert in qualitative methodology, methods seem adequate and clearly described. One of the authors performed all the interviews, which were recorded and transcribed. All four authors analysed and interpreted the content of the interviews.

In the present paper, authors report symptoms described by the patients that are in line with textbook descriptions of Graves' disease: irritability, sweating, inpatience, etc. Regarding patients' interaction with health care, mixed experiences are described. Some patients were satisfied, whereas others did not feel listened to. Patients pften lacked precise, accurate and detailed information on the disease and a clear management plan. Consistent follow-up with the same health care person was not alwyas provided, but desired by patients. Regarding the effect of the disease on patients' lives, patients described limitations in their social and personal roles. Family was important for coping.

The authors conclude that a more patient-centered approach would be useful when managing patients with Graves' disease. They also conclude that PROMS, patient reported outcome measures, would be beneficial. I am uncertain whether both, and especially the latte, are supported by the data.

I think this paper nicely presents lived experiences from patients with newly diagnosed Graves' disease. I do not have any specific comments or suggestions that could improve the paper, apart from the fact that I think that conclusions maybe are too far-reaching.

However, although there are few qualitative, interview studies on Graves, I am uncertain to what novel information this paper presents, and to what extent the experiences at the specific institution are generalizable.

Symptoms of Graves' disease have been well described previously, so in this regard the paper does not present very much new knowledge. Further, that patients should be heard, not only regarding their disease, but also how the disease affects them as persons and their lives, should by now go without saying. It is also well known that most patients benefit from seeing the same provider. That a clear and concise treatment plan should be formulated and communicated to the patient should also be standard. All these aspects: listening to and supporting the patients emotions, ensuring continuity among health care professionals caring for the patient, and providing the patient with a clear, preferrably written management plan, are universal and apply to patients with all kinds of disease. Hence, although a nicely written and performed qualitative study on patients with newly diagnosed Graves disease, and reinforcing what is already known about this disease and how health care should take care of patients today, I am uncertain to what extent this paper actually adds new knowledge.

**Reviewer**	**4**
**Name**	**Vennik, Jane**

**Affiliation**	**University of Southamption , Primary care and Population Sciences**
**Date**	**23-Sep-2025**
**COI**	**None**

Thank you for the opportunity to review this paper from a methodological perspective. Overall it was clearly written and easy to follow. The researchers used appropriate qualitative methods to explore the experiences of newly diagnosed patients with Graves Disease. The methods are well-described, and the findings appear plausible. The discussion sets the findings in the current context of what is known about this topic area.

Minor comments for consideration:

1. Would be helpful to describe how the interview topic guide was developed and whether it was piloted prior to the interviews. Did the topic guide remain the same throughout or was it developed/adapted as the interviews progressed?

2. It was clear that the interviews were conducted in Swedish, but it would be helpful to understand when the translations occurred (i.e. for the quotations). Was the coding and analysis conducted in Swedish, and then the quotations translated – or were the interviews translated and the coding in English? Who did the translation?

3. Purposeful sampling was based on age, gender, diagnosis, but there was no comment on ethnicity. It would be helpful to report this information, and then comment on the transferability of findings to other communities.

4. The list of strengths and weaknesses has been given, but would have been helpful to include some discussion about the limitations may have affected the findings/conclusions drawn from the study.

5. There is discussion about the use of PROMs for patients with GD. This could be linked better to the findings.

VERSION 1 - AUTHOR RESPONSE

Reviewer 1

Comment 1: *Concerned about the number of participants, which is relatively small.*

Answer:
Thank you for your comment. In qualitative research, sample sizes are typically smaller, as the focus is on the depth and richness of the data rather than on numerical generalizability. In our study, interviews were conducted until the research question was considered answered, meaning that no new significant information emerged from additional interviews. The sample size in our study is comparable to other qualitative studies, and we consider it appropriate and consistent with established qualitative research methodology. We have elaborated on this point in the Limitations section, lines 106-108 and 533-536.

Reviewer 2

Comment 1: *Was there anything in the study findings that were felt to be surprising? Could the study conclusions have been predicted / written before the study started?*

Answer:
Thanks for an interesting reflection. Some findings were surprising, particularly the extent to which GD affected patients' personalities and daily lives—beyond what might have been expected. While we often assume that everyone has a supportive social network, this is not always the case, highlighting the importance of addressing these issues and providing follow-up in healthcare. Furthermore, the way patients perceive their symptoms and how these symptoms influence their everyday lives emerged as a crucial factor to consider during clinical encounters. This underscores the need for healthcare professionals to actively explore patients' symptom experiences as part of a person-centred approach. We have now added more information in the discussion, lines 439-440, 441-446.

Comment 2: *Variation in empathy among health professionals is to be expected – are authors surprised?*

Answer:
We agree that this is to be expected, but our participants emphasized how strongly these variations affected their early experiences of the disease. We have clarified this nuance in the results, line 284.

Comment 3: *The fact that health professionals should be patient-centred is not revolutionary and a key component of best practice.*

Answer:
We agree that patient-centred care is already an established principle of best practice. However, our findings show that, despite this, person-centred approaches are not always consistently implemented in everyday clinical encounters. The patients' narratives highlight how a lack of person-centredness can have tangible effects on trust, understanding, and adherence early in the disease trajectory.

Comment 4: *To what extent might disease severity/biochemistry affect patient engagement?*

Answer:
Thank you for this valuable reflection. We recognize that elevated hormone levels may indeed influence how patients are able to engage with healthcare professionals, and this is an aspect we wish to better understand through the study. Our intention is to explore how such experiences shape patients' interactions and, importantly, how we might provide the right support during times when hormone levels are elevated. In this way, we hope to contribute to making it easier for patients to navigate the healthcare system and to feel more secure. We have clarified this point further in the methods section, lines 162-166.

Comment 5: *Different treatment stages at the time of interview – how does this affect interpretation?*

Answer:
Thank you for this comment. As described in the methods section, we chose to include patients at an early stage of their disease, as this represents a particularly sensitive phase during which supportive interventions may have the greatest impact. Since

qualitative interviews aim to capture the depth and variation of patient experiences, it was important to include individuals in different states in the early phase. How patients perceive their symptoms and daily life during different hormonal phases provides valuable insights into how healthcare professionals can best support them when they are in those specific states. We have added this clarification at lines 162–166.

Comment 6: *Is it realistic to fully understand the disease within 3 months?*

Answer:
As noted previously, our goal was not for patients to fully understand their disease within the first three months. Nevertheless, this period provides valuable insight into patients' initial experiences and support needs, and it was interesting to observe that some patients had already begun to find ways to manage certain aspects of their illness in a thoughtful and proactive manner. We have now added information about this in the methods/discussion sections at lines 162–166 and 454-456. During this period, patients are particularly vulnerable due to their current or recent hyperthyroidism. Qualitative data on patients in mean 6 years after diagnosis has already been published Nexö et al. Here we focus on the period where we can change care if needed.

Comment 7: *Importance of advice and guidance between consultations?*

Answer:
The interviews showed that patients often felt insecure between visits regarding medication, test results, and general well-being. Additional follow-ups can therefore provide reassurance, support patients in managing their treatment, and strengthen continuity of care. This has now been clarified in the discussion, lines 454-.

Comment 8: *Would follow-up interviews at 6 or 12 months provide more meaningful data?*

Answer:
Thank you for this thoughtful suggestion. We agree that follow-up interviews at later stages could provide valuable insights into how patients' perceptions and relationships with healthcare professionals evolve over time. This would complement our current findings from the early phase after diagnosis and something we plan for the future.

Reviewer 3

Comment 1: *Conclusions may be too far-reaching.*

Answer:
We thank the reviewer for this comment. We have carefully reviewed the conclusion section and clarified the message to ensure that it accurately reflects the study findings. We now emphasize that the findings provide insights into patients' experiences in the early stages of Graves' disease and highlight possible ways to strengthen person-centered care, lines 546-557.

Comment 2: *The study may not provide much new knowledge, and generalisability is limited.*

Answer:
Thank you for this comment. While qualitative studies do not aim for generalisability, our study provides novel insights into patients' experiences during the early phase after Graves' disease diagnosis. The findings highlight unmet needs and patient perspectives that can inform improvements in person-centered care, including the potential use of PROMs and PREMs. Although context-specific factors may influence experiences, the consistent patterns across participants suggest that the findings are transferable to other clinical settings with similar patient populations, helping healthcare providers understand and support newly diagnosed patients more effectively. This has been clarified in the manuscript, lines 534-543 and 546-557.

Reviewer 4

Comment 1: *Would be helpful to describe how the interview topic guide was developed and whether it was piloted prior to the interviews. Did the topic guide remain the same throughout or was it developed/adapted as the interviews progressed?*

Answer:
We worked together with a patient when we designed the study guide to include relevant perspectives. Our aims were to evaluate the experiences of the disease from patients' perspective and what support that was needed. After we had a prototype of the guide, we tested it on some patients and modified it further. and identify who participated and gave their opinion on the questions. In Lines 179-182 it, we have clarified in that section that the interview guide was also developed in collaboration with the patient representative in line 191 and that we tested in some patients before starting the first interview.

Comment 2: *It was clear that the interviews were conducted in Swedish, but it would be helpful to understand when the translations occurred (i.e. for the quotations). Was the coding and analysis conducted in Swedish, and then the quotations translated – or were the interviews translated and the coding in English? Who did the translation?*

Answer:
Thank you for your comment, we have clarified how the process was done in the article under the data analysis section in lines, 206-209.

Comment 3: *Purposeful sampling was based on age, gender, diagnosis, but there was no comment on ethnicity. It would be helpful to report this information and then comment on the transferability of findings to other communities.*

Answer:
Thank you for drawing attention to this issue and we have clarified this under the section participants and setting, lines 155 and 170.

Comment 4: *The list of strengths and weaknesses has been given but would have been helpful to include some discussion about the limitations may have affected the findings/conclusions drawn from the study.*

Answer:
We thank the reviewer for their helpful comments. In response, we have clarified the first section with a bullet-point list of strengths and weaknesses, line 93-108, and added a
paragraph discussing the methodological limitations, line 533-543.

Comment 5: *There is discussion about the use of PROMs for patients with GD. This could be linked better to the findings.*

Answer:
Thank you for pointing this out. We agree that the connection needed to be clarified. We have now added a section explaining how PROMs and PREMs can be linked to care and how they may help patients, line 521-524 and 552-555.
